# Algorithm of OMA for large-scale orthology inference

**DOI:** 10.1186/1471-2105-9-518

**Published:** 2008-12-04

**Authors:** Alexander CJ Roth, Gaston H Gonnet, Christophe Dessimoz

**Affiliations:** 1 ETH Zurich, and Swiss Institute of Bioinformatics, 8092 Zurich, Switzerland

## Abstract

**Background:**

OMA is a project that aims to identify orthologs within publicly available, complete genomes. With 657 genomes analyzed to date, OMA is one of the largest projects of its kind.

**Results:**

The algorithm of OMA improves upon standard bidirectional best-hit approach in several respects: it uses evolutionary distances instead of scores, considers distance inference uncertainty, includes many-to-many orthologous relations, and accounts for differential gene losses. Herein, we describe in detail the algorithm for inference of orthology and provide the rationale for parameter selection through multiple tests.

**Conclusion:**

OMA contains several novel improvement ideas for orthology inference and provides a unique dataset of large-scale orthology assignments.

## Background

The classification of genes according to evolutionary relations is essential for many aspects of comparative and functional genomics. Evolutionary relations are often described as pairwise relations. Two genes that share a common ancestor are defined as homologs, while genes that are similar in sequence without a common origin are termed analogs. Homologs can be divided into several classes [1]: orthologs, which originate from a speciation event; paralogs, which originate from gene duplication; and xenologs, which originate from horizontal gene transfer. Orthologs are valuable in numerous analyses, including reconstruction of species phylogenies, protein function inference, database annotation, and genomic context analysis.

Evolutionary relations can also be defined with respect to a third gene. Paralogs are classified as out-paralogs or in-paralogs [2]. In-paralogs are genes that diverged by a duplication that occurred *after *a speciation event of reference. The term co-orthologs is used occasionally to describe the same scenario from the perspective of a third gene that is orthologous to both genes. In contrast, out-paralogs are paralogs that diverged *before *a particular speciation event of reference.

To address the need for reliable sources of orthologs, several initiatives have been created for better orthologs prediction Commonly, there are two classes of prediction methods: *phylogeny *based methods, which compare gene trees with species trees (e.g. NOTUNG [3], Orthostrapper [4], RIO [5], Softparsmap [6], LOFT [7], Ensembl [8]) and *pairwise *based methods, which perform homology search with (optional) subsequent clustering (e.g. BBH [9], COG [10], InParanoid [11], KOG [12], OrthoMCL [13], RSD [14], MultiParanoid [15], Roundup [16], Homologene [17], eggNOG [18]).

In 2005, we introduced the OMA orthology prediction project with the goal to classify all orthologs in completely sequenced genomes [19]. OMA is a pairwise based method with a number of distinctive features: alignments are performed using an efficient implementation of full Smith-Waterman dynamic programming [20] (as opposed to methods with lower sensitivity such as BLAST), confidence intervals explicitly consider estimation uncertainty, and exclusion of paralogs is achieved using sequences in third-party genomes as "witnesses of non-orthology" [21].

Since then, we have substantially improved the OMA algorithm. Orthology is now inferred on the basis of evolutionary distances rather than alignment scores, the predicted orthologs are no longer limited to one-to-one orthologs. We build groups of orthologs using a maximum-weight clique algorithm. A web interface now enables interactive exploration of the predictions [22]. In addition, the number of complete genomes under analysis has increased to over 657, which requires efficient solutions regarding computation speed and memory consumption.

In this paper, we describe the current OMA algorithm in detail, motivate our parameter selection, and offer a discussion about the method and results.

## Methods

**Figure 1:**
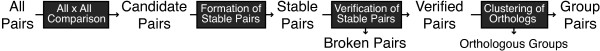
**Algorithm flow chart**. Boxes represent the steps of the algorithm, and arrows are the input and output data for each step.

The algorithm of OMA takes as input a set of complete genomes and outputs pairs of orthologous genes that are optionally clustered into orthologous groups. The algorithm follows four steps (see Figure 1):

**Step 1**: To find homology, we compute pairwise alignments between *all pairs *of sequences for all genes in all genomes. Pairs with significant alignment scores are retained as *candidate pairs*.

**Step 2**: Orthologs are usually the closest genes in two genomes, because they started diverging at speciation, whereas paralogs started diverging at a duplication prior to speciation. Genes across genomes that are mutually the most closely related sequences, taking into account inference uncertainty, are upgraded to *stable pairs*.

**Step 3**: In cases where an ortholog is missing, we seek to avoid erroneous classification of paralogs as orthologs (pseudo-orthologs) by verifying stable pairs with sequences in a third genome that can act as witness of evolution. Pairs that pass the verification step are upgraded to *verified pairs*, and pairs that do not pass are paralogs and referred to as *broken pairs*.

**Step 4:** For some applications, such as species tree reconstruction, it is advantageous to cluster orthologs into *orthologous groups*. Pairs of sequences in such groups are termed *group pairs*.

In the following, we describe each of the four steps, and motivate all parameter choices.

### All-against-all alignments

The goal of the first step of the process is homology detection. All pairs of protein sequences from complete genomes are aligned using full dynamic programming. There are several advantages of using protein sequences rather than using DNA sequences. Very distant homologies are difficult to find at the DNA level, and protein sequences suffer less from convergence due to mutational biases. Also, the length of a protein is one third of that of the corresponding DNA sequence, a considerable advantage given that the time complexity of aligning sequences is quadratic with respect to length. The disadvantage to using protein sequences instead of DNA is that complications arising from multiple gene products must be handled explicitly by selecting the longest splice variant as well as isoforms with at least 10% non-redundant positions. The sequences used by OMA are from public databases (mainly Genbank [23] for Prokaryotes and Ensembl [8] for Eukaryotes) and all data are checked for consistency and quality.

Homology is established in two sub-steps. First, alignments between all sequences are performed using full, local dynamic programming with a fixed PAM matrix to find all homologous sequences [20,24]. Second, significant alignments (score > 85) are refined by searching among all PAM distances the scoring matrix that maximizes the alignment score. Since scores are the log of odd ratios, the PAM number of this matrix corresponds to the maximum likelihood of evolutionary distance. Empirically, we observed that with a mixture of homologous and non-homologous pairs of sequences as input, the PAM-224 matrix yields alignment scores that are on average closest to the ones obtained in the refinement part. Thus, this is the fixed matrix that we use in the first part. Refined alignments with scores above 181 (which roughly corresponds to an E-value of 10^-14^) are considered significant. With scores below this value, the proportion of candidate pairs that end up being predicted as orthologs decreases rapidly (data not shown).

The all-against-all step is computationally expensive, and the run time increases quadratically with the total number of amino acids in a protein sequence. The use of a heuristic-based algorithm such as BLAST could potentially increase the speed of the homology search, but modern implementations of Smith-Waterman using SIMD instructions are almost as fast as BLAST [25]. Moreover, most of the time is consumed by estimating evolutionary distances.

Since we consider entire proteins as the basic evolutionary unit, why then not use global alignments? Protein ends are often variable, and thus, it is reasonable to ignore them by using local alignments. To guarantee that a significant fraction of a sequence is aligned, we use a *length tolerance *criterion. The length of the shorter aligned sequence must be at least the fraction ℓ of the longest sequence. That is

min (|*a*_1_|, |*a*_2_|) > ℓ·max(|*s*_1_|, |*s*_2_|)

where *a*_1 _and *a*_2 _are the lengths of the aligned subsequences of *s*_1 _and *s*_2_. Alignments that pass both the length and score criteria are upgraded to *candidate pairs *(CP).

#### Parameter selection and validation

The parameter ℓ is determined by two tests. The first test, the *triangle inequality test*, is performed over all candidate pairs. Under a time-reversible Markovian model, the evolutionary distances between homologous sequences should obey the triangle inequality condition which requires that in a triplet of sequences, any distance between two sequences be less than or equal to the sum of the other two distances. Because these distances are estimates, this property is expected only to hold within a confidence interval.

*d*_xz _≤ *d*_xy _+ *d*_xz_

**Figure 2:**
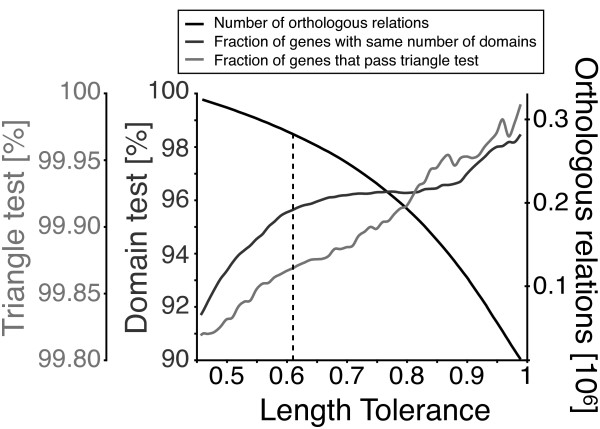
**Tests to determine the optimum length criterion value**. The fraction of candidate pairs that pass the triangle inequality test and the fraction that have the same number of domains increases with stricter (higher) length tolerance. In contrast, the number of predicted orthologous relationships decreases with stricter length tolerance. To consider only alignments that cover at least a fraction of the sequence length, a length criterion value of ℓ = 0.61 is used in this study.

For example, this condition may not be met if the sequences x and y share one domain and sequences y and z share another domain, but sequences x and z are not related. The triangle inequality test detects such violations, which are likely to arise when inconsistent sequences parts are matched. With increasing (i.e. stricter) length criteria, a larger fraction of candidate pairs pass the triangle inequality test (Figure 2).

Many proteins consists of several domains originating from gene fusions, deletions, and internal repetitions. The majority of multi-domain proteins have evolved by the stepwise insertions of single domains [26]. In the second test, candidate pairs are verified by testing the assumption that the number of domains for orthologous sequences are in agreement, including identical domains (e.g. repetitions). Domain information is obtained from the Pfam database and consists of conserved protein regions and domains [27]. The amount of proteins with the same number of domains increase with stricter length tolerance, but a "plateau" is observed for 0.6 < ℓ < 0.9 (Figure 2).

Figure 2 shows the results of the two validation tests and also that the number of orthologous relations (i.e. VP) decreases with increasing length criteria. A trade-off exists between sensitivity and selectivity. A value of ℓ = 0.61 is a good compromise between minimizing triangle inequality violations and numbers of different domains while still including enough ambiguous alignments.

### Formation of stable pairs

In the second step of the algorithm, potential orthologs are detected by the identification of sequences in two genomes that are more closely related to each other than to any other sequence in the other sequence. We term these sequences *stable pairs *(SP). This name was chosen due to its close association with the stable marriage problem in computer science.

To measure the relatedness of sequences, either similarity scores or evolutionary distances can be used. Most methods employ the similarity score ("best hit"), because it is directly obtained by the alignment process and the highest scoring sequence is usually the most closely related sequence. However, scores do not constitute a direct measure of relatedness. In particular, they depend on protein lengths. Evolutionary distances such as PAM units, though more expensive to compute, constitute a sounder measure of relatedness, because distances are additive in their expected value (i.e. they are expected to equal the sums of branch lengths between the species) and have well characterized statistical properties.

A tolerance interval is used to allow the inclusion of more than one potential ortholog, as this becomes necessary when a gene duplication event occurred after speciation. The tolerance threshold can be defined by including similarity scores in an interval below the top score, or by using variance of distance estimates to compute a confidence interval.

**Figure 3:**
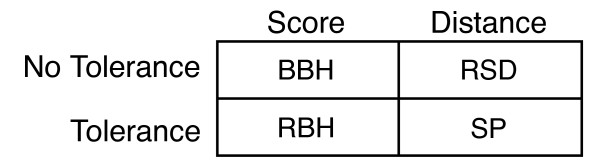
**Different methods to find potential orthologs**. The mutual best alignment can be determined by similarity score or by evolutionary distance (columns), and with or without the use of a tolerance to include multiple orthologous relationships (rows).

Consequently, orthology assignment methods based on pairwise sequence comparison can be classified in four categories (see Figure 3). Bidirectional best hits (BBH) is the most common approach and uses scores with no tolerance (e.g. [12]). Reciprocal-best-BLAST-hits (RBH) is based on BLAST scores and uses a tolerance by including all hits within a p-value [28]. The reciprocal smallest distance algorithm (RSD) use evolutionary distances without a tolerance [14,16]. The stable pair method of OMA use distance to measure relatedness between genes and their variances as a tolerance criterion.

As mentioned above, the use of confidence intervals is necessary to account for many-to-many orthologous relations, which arise when duplications occurred after speciation. Additionally, distance estimation is subject to inference uncertainty and, thus, true orthologs may not have the shortest estimated distance.

Formally, a pair of sequences (x, y) from genomes X and Y is considered a stable pair if and only if, for all x_*i *_∈ X, x_*i *_≠ x, and for all y_*j *_∈ Y, y_*j *_≠ y:

$$d_{{\text{xy}}_{j}}-d_{\text{xy}}>k\sqrt{\sigma^{2}(d_{{\text{xy}}_{j}}-d_{\text{xy}})}$$

and

$$d_{{\text{x}}_{i}\text{y}}-d_{\text{xy}}>k\sqrt{\sigma^{2}(d_{{\text{x}}_{i}\text{y}}-d_{\text{xy}})}$$

where *d *is a pairwise maximum likelihood distance estimate and *k*, the tolerance parameter of the standard deviation between the two distances, where $\sigma^{2}(d_{{\text{xy}}_{j}}-d_{\text{xy}})=\sigma^{2}(d_{{\text{xy}}_{j}})+\sigma^{2}(d_{\text{xy}})-2$. An estimate of the variance is obtained by the distance estimation, while efficient estimation of covariance for this case was previously shown [21].

#### Parameter selection and validation

The tolerance parameter *k *controls the balance between sensitivity (more true orthologs as stable pairs) and selectivity (few out-paralogs as stable pairs). The optimal value of *k *for our purpose is determined using the *out-paralog test*.

**Figure 4:**
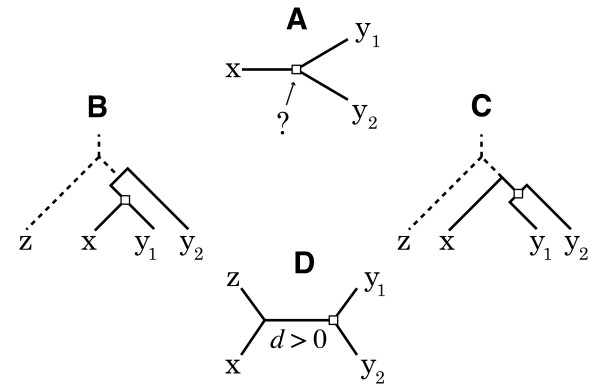
**Test to distinguish in-paralogs and out-paralogs**. **A **What is the relationship of sequences y_1 _and y_2 _with regard to x? Identify which branch to place the root by finding an out-group sequence z. **B **If the root on the branch leading to x, then y_1 _and y_2 _are out-paralogs. **C **If duplication takes place after speciation, y_1 _and y_2 _are in-paralogs. **D **To test if y_1 _and y_2 _are in-paralogs, we confirm that the distance *d *of the internal branch is greater than zero.

The out-paralog test is designed to discriminate cases of one-to-many orthology from cases of out-paralogy. More precisely, it determines whether the divergence of sequences x, y_1 _and y_2_, illustrated in Figure 4A, is due to a speciation or a duplication event. This is evaluated by finding on which branch to place the root. If the root is located on the branches leading to y_1 _or y_2_, this suggests that the divergence is a speciation and that the sequence y_2 _is an out-paralog (Figure 4B). In contrast, if the root is on the branch leading to x, the divergence is a duplication and both sequences in Y are orthologous to x (Figure 4C). To find a suitable out-group z to place the root, the information of a trusted phylogenetic topology is used (i.e. a representative phylogeny that is indisputable). The sequence z is selected to be the gene closest to x in the out-group genome Z that is closest to the divergence of X and Y. Figure 4D shows the quartet that is the result of y_1 _and y_2 _being in paralogs. If the length of the internal branch *d *for the given topology (i.e. the least square fit) is greater than zero, the sequences are accepted.

$$d=\frac{d_{{\text{zy}}_{1}}+d_{{\text{zy}}_{2}}+d_{{\text{xy}}_{1}}+d_{{\text{xy}}_{2}}-2d_{\text{zx}}-2d_{{\text{y}}_{1}{\text{y}}_{2}}}{4}>0$$

**Figure 5:**
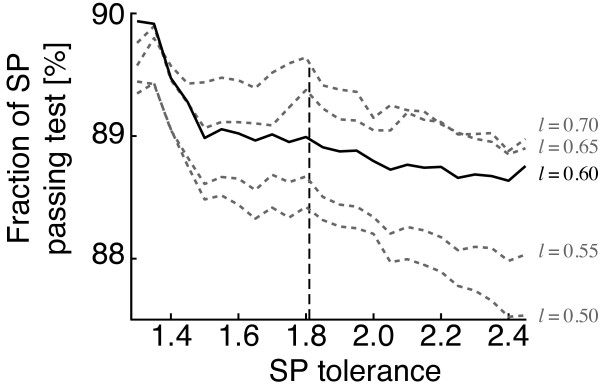
**Value for stable pair tolerance parameter**. The fraction of stable pairs that pass the out-paralog test has a local optimum at the SP-tolerance 1.81, for five different length criteria. Increasing the tolerance value results in a larger fraction of stable pairs suspected as out-paralogs.

To evaluate the parameter *k*, the fraction of SP that passes the test is measured. Figure 5 depicts the decreasing fraction of passing stable pairs with increasing stable pair tolerance at different length criteria. Again, the problem is to reduce the amount of conflicting out-paralogs while not discarding interesting many-to-many relationships. In this implementation, the required distance for a more distant stable pair must be within *k *= 1.81 of the closest stable pair.

### Verification of stable pairs

**Figure 6:**
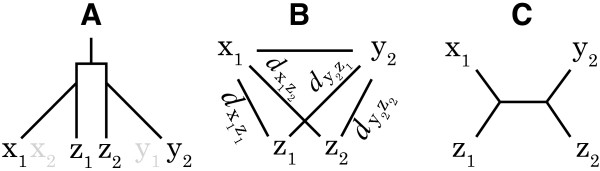
**Assignment of potential paralogs**. **A **In an evolutionary scenario, an ancestral gene is duplicated, followed by two speciation events, followed by asymmetrical gene loss of genes x_2 _and y_1_. The paralogous genes x_1 _and y_2 _could be mistaken for orthologs, but the duplicates are retained in genome Z that can act as a witness of non-orthology. **B **Schematic for verifying a stable pair between x_1 _and y_2 _using genome Z. If (x_1_, z_1_) and (y_2_, z_2_) form stable pairs and are the closest relatives then x_1 _and y_2 _are paralogs and were not verified. **C **The only possible quartet formed when (x_1_, z_1_) and (y_2_, z_2_) are the closest related sequences is shown.

Although the construction of stable pairs is likely to identify the corresponding ortholog of each sequence, at least one special case exists in which systematic failure will occur: differential gene loss. This problem affects all pairwise approaches, and is shown in Figure 6A. An ancient duplication event is followed by two speciation events resulting in three species X, Y, and Z. In two of these species, each of the duplicates is lost (e.g. x_2 _and y_1_), and as a result, when comparing species X and Y, x_1 _and y_2 _are the highest scoring match. In such a case, (*x*_1_, *y*_2_), although paralogs, form a stable pair.

The purpose of the third step is to detect such stable pairs corresponding to non-orthology. The presence of a third genome Z, which has retained both copies z_1 _and z_2 _of the duplication event, acts as a *witness of non-orthology*. We previously described the details of this procedure [29], and the idea is illustrated in Figure 6B. If $d_{{\text{x}}_{1}{\text{z}}_{1}}$ is significantly shorter than $d_{{\text{x}}_{1}{\text{z}}_{2}}$ and $d_{{\text{y}}_{2}{\text{z}}_{2}}$ is significantly shorter than $d_{{\text{y}}_{2}{\text{z}}_{1}}$, there is evidence that x_1 _and y_2 _may not be orthologs. Figure 6C depicts the most likely quartet predicted from the data provided in Figure 6A. This approach can also be viewed as a tree-reconciliation that is based on quartets without assuming any species tree topology.

Each stable pair is verified by comparison to all other genomes. Stable pairs for which no witness of non-orthology could be found are termed *verified pairs *(VP) and are likely to be orthologs. Furthermore, stable pairs that are not verified were defined as *broken pairs *(BP) and are likely to correspond to paralogs.

Such cases of differential gene losses are not uncommon in nature. Among yeasts for instance, approximately 5% of stable pairs are detected as non-orthologous using the procedure described above.

#### Parameter selection and validation

**Figure 7:**
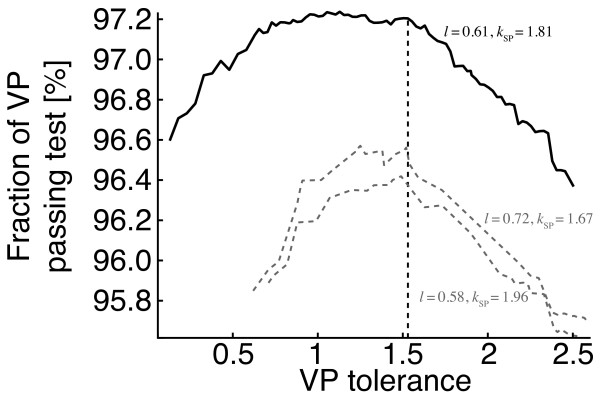
**Value for verified pair tolerance parameter**. The fraction of verified pairs that passes the out-paralog test is drawn. The top curve is produced with the use of the optimal previous parameters, and the lower curves are produced at other parameter settings and also have locally optimal values, both show similar optimal values (1.53) as the best curve.

The procedure again uses a tolerance parameter to tune the width of the confidence intervals required to detect non-orthologs. To optimize the parameter, we use the out-paralog test (described in the previous section). We select the VP tolerance value such that the fraction of verified pairs that pass the test is maximized. In figure 7, the fraction of passing VPs as a function of the VP tolerance is charted. In terms of the trade-off between VP- and the SP-tolerance, increasing the VP-tolerance has little effect when the SP-tolerance is low (i.e. only the closest stable pairs were chosen). The decrease in the amount of VPs with stricter VP-tolerance is much less than with stricter SP-tolerance. Hence, it is reasonable to have a stricter VP-tolerance than SP-tolerance to maximize coverage. From the plot, a VP-tolerance of *k *= 1.53 visibly constitutes a reasonable trade-off.

Note that although both the verification of the SP step and the out-paralogy test detect non-orthologs, the test requires knowledge of the species tree. To keep the orthology prediction independent from such (often uncertain) knowledge, we only used the out-paralogy test for parameter fitting, and only in cases where the species topology is undisputed.

### Ortholog clustering

The final step of the algorithm creates groups of orthologs. Such grouping is non-trivial, because orthology is defined over pairs of sequences and is not necessarily a transitive relation. For instance, a sequence in one genome may form several verified pairs with sequences in another genomes, corresponding to several orthologous relations (co-orthologs). These in turn cannot be orthologous to each other. In OMA, we address this problem by making available both pairwise orthologous relations (the verified pairs) and groups of genes in which all pairs are orthologs. Though the OMA groups leave out orthologous relations, they are useful for some applications, such as species tree inference.

**Figure 8:**
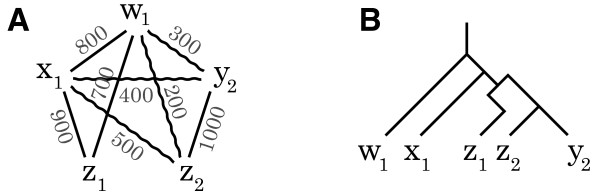
**A **An example graph containing one 4-clique, four 3-cliques, and eight 2-cliques is provided. The highest scoring partition of the graph is {w_1_, x_1_, z_1_}, {y_2_, z_2_}. **B** A possible evolutionary scenario corresponding to the graph.

We use a clique algorithm to search for maximal, completely connected subgraphs in a graph, where the vertices are genes and the edges are verified pairs. To compute cliques, algorithms exist to maximize either the size of the clique (number of vertices) or the total weight of cliques (sum of edge weights). Figure 8A shows a graph with edges between all vertices except (z_1_, z_2_) and (z_1_, y_2_), which are paralogous relations. The highest scoring partition is {w_1_, x_1_, z_1_}, {y_2_, z_2_}, with the total sum of edge weights of 700 + 800 + 900 + 1000 = 3600. The score is higher than the highest scoring maximum size clique {w_1_, x_1_, y_2_, z_2_}, {z_1_}, where the sum of the scores is 200+300+400+500+800+1000 = 3200. Hence, a smaller clique is chosen due to higher edge weights, which correctly assigns orthologs according to the hypothetical evolutionary scenario in Figure 8B, where the duplication give subscripts that correspond to functionality. Finding cliques is a NP-complete problem and the implementations used here are based on an approximation of the vertex cover problem [30]. Each clique constitutes an *orthologous group*, where the sequence pairs in an orthologous group are denoted *group pairs *(GP), corresponding to close orthologs.

#### Parameter selection and validation

To validate our methods and to compare different algorithms for clique construction, a species tree is built from the orthologous groups produced by each algorithm, and the fit of the data to the tree is measured using the dimensionless index [31]. This technique assumes that if the groups inferred by the clique algorithms correctly predict orthology, the data will have a better fit to the species tree.

For verification, 100 trials using various genomes and different taxa are computed using four different clique versions. *Maximum size clique *chose the largest clique in the graph starting with the highest scoring edge (but does not use any other edge information). *Maximum size score clique *is an extension that uses the sum of the edge weights and selects the higher scoring clique from several maximum cliques of same size. The above described algorithm, *maximum edge weight clique*, is used twice, first with the *scores *and then with the *distances complement *as edge weights.

In general, the maximum edge weight clique algorithms perform better than the maximum size cliques (in 82 % of the trials), as shown in Table 1. This result supports the argument of the hypothetical example in Figure 8. To build maximum edge weight clique we chose scores as edge weights, over distances, because scores provides better fits of the data to the constructed species trees twice as often.

**Table 1:** Results of clustering

**Clique**	**Times best**
Maximum size	1
Maximum size score	17

Maximum edge weight (distance)	28
Maximum edge weight (score)	**54**

Evaluation of the quality of the trees built from orthologous groups using different clustering methods. The quality of trees is measured using the dimensionless index [31].

## Results and discussion

### Assigning evolutionary relationships

**Figure 9:**
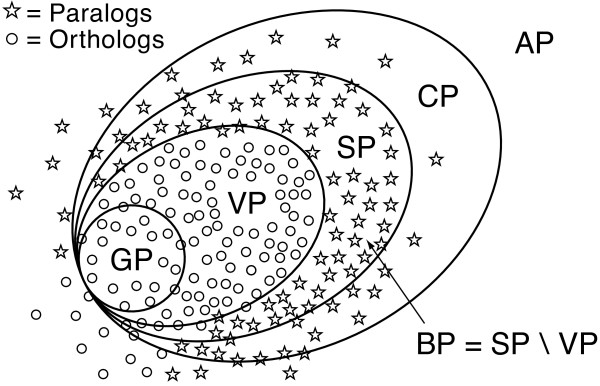
**Evolutionary relations and corresponding classes of pairs**. The hierarchy of pairs are classified according to evolutionary relations. We seek to find the borders of pairs to capture underlying evolutionary relations. Verified pairs are designed to cover all orthologs, and group pairs are a subset of the closest orthologs. Broken pairs are cases where paralogy is explicitly classified.

**Figure 10:**
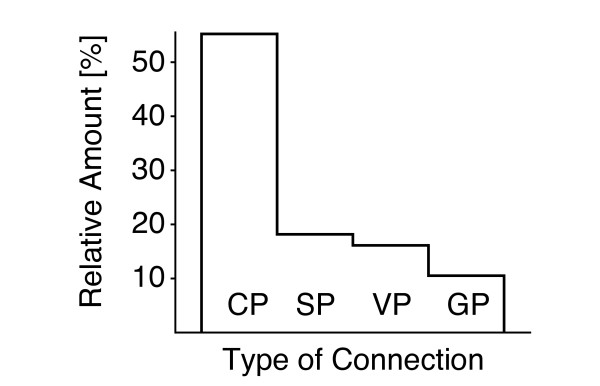
**Number of pairs reported after each step**. Each step of the algorithm reduces the number of pairs, and the largest reduction is observed with the formation of stable pairs.

The goal of the OMA project is to detect all orthologous sequence relationships among sequenced genomes. Considering that orthology is a pairwise relation, the starting point is all $\left(\begin{array}{c} n\\ 2 \end{array}\right)$ sequence pairs that successively filter through several steps to yield pairs of orthology (Figure 9). Table 2 lists the names of shrinking subsets and their meaning in terms of the corresponding evolutionary relationships. The first step of the method, the all-against-all alignment, removes the majority of pairs and leaves only a small fraction of candidate pairs. These are further filtered in subsequent steps, and the decrease of the relative number of pairs after each step is depicted in Figure 10. A substantial reduction occurs with the classification of stable pairs from candidate pairs and serves to reduce the complexity of the verification step. The reduction of stable pairs to verified pairs is comparatively small, but nevertheless important, since this crucial step removes non-orthologs and explicitly indicates cases of paralogy (broken pairs). Finally, group pairs are the fewest in number, because all but the most similar orthologous relationships have been removed.

**Table 2:** Sequence pairs and their corresponding evolutionary relationships

**Pairs**	**Evolutionary Relation**
All pairs (AP)	Any
Candidate pairs (CP)	Homologs
Stable pairs (SP)	Orthologs, Pseudo-orthologs
Broken pairs (BP)	Paralogs
Verified pairs (VP)	Orthologs
Group pairs (GP)	Close orthologs

### Verified pairs

Verified pairs represent a useful resource that describe many-to-many orthology while pseudo-orthologs from differential gene loss have been removed. In other words, the most similar sequences may not be orthologous, and for this reason, all stable pairs are verified using a third genome as a witness of non-orthology. A critical assumption in the verification of stable pairs is that in at least some genomes both copies of a duplication event are present. It is possible that no duplicates remain and that paralogy cannot be detected by sequence similarity. However, the increasing number of completed genomes also increases the chance of observing duplicates in a genome. Both paralogs are often present in multiple genomes. For example, when predicting orthologs for the subset of Firmicutes, (the subset is used for computational reasons) 75% of broken pairs had more than one witness of non-orthology.

Lateral gene transfer (LGT) events of homologous sequences (xenologs) are difficult to distinguish from duplication events. Two genes may appear to be duplicates when in fact, they are not. This issue affects most orthology prediction methods. In the case of OMA, the verification step helps reducing the adverse impact of LGT; furthermore, we are investigating reliable ways of excluding the most obvious cases of LGT.

### Fusion-fission events

Two genes that in one organism may be truly orthologous to one fused genes in another organism. OMA considers the entire protein, rather than domains, to be the basic evolutionary unit. Users interested in gene fusion-fission events, or in domain evolution, may view this as a limitation. We have chosen to exclude such scenarios, due to the difficulty to separate these events from the cases where the domains in proteins arose form different evolutionary unrelated domains. Although part of the sequence may have diverged through speciation, another part is clearly non-homologous. If orthology is defined at the domain level, a gene could be orthologous to two or more sequences that are completely unrelated. In terms of function, which is often inferred from orthology, genes with different domains are unlikely to be similar. Finally, restricting potential orthology to genes with a majority of homologous positions presents the advantage of not only avoiding these problems, but also reducing computational complexity.

### Orthologous groups

In OMA, orthologous groups consist of close orthologs, which are useful to build species trees. The results of grouping close orthologs is represented by an ortholog matrix. In this matrix, rows correspond to groups of orthologous genes, and columns correspond to genomes. A non-empty element in M_*i*,*j *_indicates that a genome *j *has a member in an orthologous group *i*. The members of a group possess at most one close ortholog in each genome.

In cases where duplication events occurred after a speciation event, several orthologous relationships exist and are often referred to as co-orthologs or in-paralogs [2]. We group orthologs such that the most similar protein sequence belong together using maximum edge weight cliques. It should be noted that the most similar sequences do not necessarily have the most similar function [32].

Using cliques to construct groups is a strict requirement, because if an edge is missing (due to weak similarity or misclassification), the group gets split. For applications where this is problematic, users can devise their own grouping strategy from the orthologous pairs, which we also make available.

**Figure 11:**
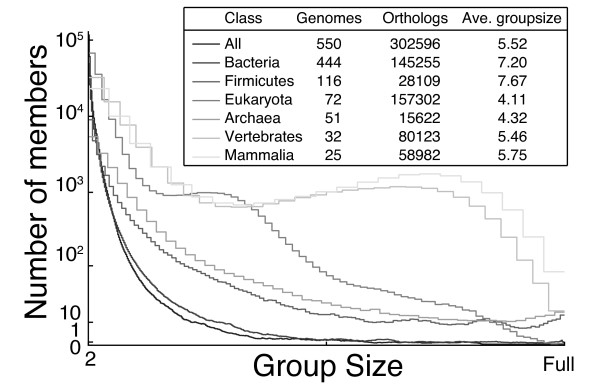
**Distribution of group size**. The average group size is drawn for several versions of orthologous matrices. For large sets of genomes (e.g. All and Bacteria) very few groups are full (i.e. have one member from each genome).

The distribution of group sizes for different sets of genomes is displayed in Figure 11. The average size of the orthologous groups (number of genes per group) is relatively small in comparison to other methods (ranging from approximately 4 to 7 genes per group). More small groups exist rather than large groups, which is expected based on the occurrence of duplications throughout evolution. Large groups commonly consist of highly conserved genes, such as ribosomal proteins.

### Exhaustive sequence alignments

The all-against-all step is computationally expensive, and the time complexity is *O*((*n*_1 _+ *n*_2 _+ ... + *n*_*k*_)^2^) where *n*_*k *_represents the number of sequences in the *k*^th ^genome. As of November 2008, we have computed nearly 6 trillion sequence alignments. A total of approximately 12 Hexaflop or roughly 500 years of CPU time. Of these alignments, 3.2 billion were considered significant (i.e. score > 130). This dataset constitutes a valuable resource for comparative studies and is available upon request. The subsequent steps of the algorithm are comparatively fast.

### Comparison to other projects

The performances of OMA are compared to other projects in a separate article [33]. The study includes COG, KOG, EggNOG, InParanoid, OrthoMCL, Ensembl, Homologene, and RoundUp. The study tests ortholog predictions on the basis of phylogeny (through reconstruction of orthologous gene trees and through comparison with phylogenetic analyses from the literature) and on the basis of function conservation (in terms of GO annotation, EC number classification, expression level, and gene neighborhood conservation). The results of OMA are among the best in the phylogenetic tests. In functional tests, it also performs well where high functional specificity is required, at the expense of a lower recall than projects such as OrthoMCL or EggNOG.

In terms of size and with 657 genomes analyzed, OMA is by a wide margin the largest orthologs inference effort (the second largest, EggNOG, includes 373 genomes). Our website is regularly updated as new species get included.

## Conclusion

Orthology is interesting for a wide range of bioinformatics analyses, including functional annotation, phylogenetic inference, or genome evolution. This paper describes and motivates the algorithm of OMA for predicting orthologous relationships among complete genomes. The algorithm takes a pairwise approach, thus neither requiring tree reconstruction nor reconciliation, and offers the following improvements over the standard bidirectional best hit approach: i) the use of evolutionary distance instead of score, ii) a tolerance that allows the inclusion of one-to-many and many-to-many orthologs, iii) consideration of uncertainty in distance estimations, iv) detection of potential differential gene losses. The algorithm is characterized by four parameters that are optimized using independent tests. The current status of the project and the project results, including phylogenetic trees derived from the data, are available online [34].

## Authors' contributions

GG initiated the project, devised and implemented the algorithm. Later refinements were performed by all three authors. AR and CD designed the tests for parameter selection. AR implemented the tests and performed the analyses. AR and CD wrote the manuscript.

## References

[B1] Fitch WM (1970). Distinguishing homologous from analogous proteins. Syst Zool.

[B2] Sonnhammer ELL, Koonin EV (2002). Orthology, paralogy and proposed classification for paralog subtypes. Trends Genet.

[B3] Chen K, Durand D, Farach-Colton M (2000). NOTUNG: a program for dating gene duplications and optimizing gene family trees. J Comput Biol.

[B4] Storm CEV, Sonnhammer ELL (2002). Automated ortholog inference from phylogenetic trees and calculation of orthology reliability. Bioinformatics.

[B5] Zmasek CM, Eddy SR (2002). RIO: analyzing proteomes by automated phylogenomics using resampled inference of orthologs. BMC Bioinformatics.

[B6] Berglund-Sonnhammer AC, Steffansson P, Betts MJ, Liberles DA (2006). Optimal gene trees from sequences and species trees using a soft interpretation of parsimony. J Mol Evol.

[B7] Heijden RTJM van der, Snel B, van Noort V, Huynen MA (2007). Orthology prediction at scalable resolution by phylogenetic tree analysis. BMC Bioinformatics.

[B8] Flicek P, Aken B, Beal K, Ballester B, Caccamo M, Chen Y, Clarke L, Coates G, Cunningham F, Cutts T, Down T, Dyer S, Eyre T, Fitzgerald S, Fernandez-Banet J, Graf S, Haider S, Hammond M, Holland R, Howe K, Howe K, Johnson N, Jenkinson A, Kahari A, Keefe D, Kokocinski F, Kulesha E, Lawson D, Longden I, Megy K, Meidl P, Overduin B, Parker A, Pritchard B, Prlic A, Rice S, Rios D, Schuster M, Sealy I, Slater G, Smedley D, Spudich G, Trevanion S, Vilella A, Vogel J, White S, Wood M, Bir-ney E, Cox T, Curwen V, Durbin R, Fernandez-Suarez X, Herrero J, Hubbard T, Kasprzyk A, Proctor G, Smith J, Ureta-Vidal A, Searle S (2007). Ensembl 2008. Nucleic Acids Res.

[B9] Mushegian AR, Koonin EV (1996). A minimal gene set for cellular life derived by comparison of complete bacterial genomes. Proc Natl Acad Sci USA.

[B10] Tatusov RL, Koonin EV, Lipman DJ (1997). A genomic perspective on protein families. Science.

[B11] Remm M, Storm CE, Sonnhammer EL (2001). Automatic clustering of orthologs and in-paralogs from pairwise species comparisons. J Mol Biol.

[B12] Tatusov RL, Fedorova ND, Jackson JD, Jacobs AR, Kiryutin B, Koonin EV, Krylov DM, Mazumder R, Mekhedov SL, Nikolskaya AN, Rao BS, Smirnov S, Sverdlov AV, Vasudevan S, Wolf YI, Yin JJ, Natale DA (2003). The COG database: an updated version includes eukaryotes. BMC Bioinformatics.

[B13] Li L, Stoeckert CJJ, Roos DS (2003). OrthoMCL: identification of ortholog groups for eukaryotic genomes. Genome Res.

[B14] Wall DP, Fraser HB, Hirsh AE (2003). Detecting putative orthologs. Bioinformatics.

[B15] Alexeyenko A, Tamas I, Liu G, Sonnhammer ELL (2006). Automatic clustering of orthologs and inparalogs shared by multiple proteomes. Bioinformatics.

[B16] DeLuca TF, Wu IH, Pu J, Monaghan T, Peshkin L, Singh S, Wall DP (2006). Roundup: a multi-genome repository of orthologs and evolutionary distances. Bioinformatics.

[B17] Wheeler DL, Barrett T, Benson DA, Bryant SH, Canese K, Chetvernin V, Church DM, DiCuccio M, Edgar R, Federhen S, Geer LY, Kapustin Y, Khovayko O, Landsman D, Lipman DJ, Madden TL, Maglott DR, Ostell J, Miller V, Pruitt KD, Schuler GD, Sequeira E, Sherry ST, Sirotkin K, Souvorov A, Starchenko G, Tatusov RL, Tatusova TA, Wagner L, Yaschenko E (2007). Database resources of the National Center for Biotechnology Information. Nucleic Acids Res.

[B18] Jensen L, Julien P, Kuhn M, von Mering C, Muller J, Doerks T, Bork P (2007). eggNOG: automated construction and annotation of orthologous groups of genes. Nucleic Acids Res.

[B19] Dessimoz C, Cannarozzi G, Gil M, Margadant D, Roth A, Schneider A, Gonnet G, McLysath A, Huson D (2005). OMA, A Comprehensive, Automated Project for the Identification of Orthologs from Complete Genome Data: Introduction and First Achievements. RECOMB 2005 Workshop on Comparative Genomics, Volume "LNBI3678" of Lecture Notes in Bioinformatics.

[B20] Smith TF, Waterman MS (1981). Identification of common molecular subsequences. J Mol Biol.

[B21] Dessimoz C, Gil M, Schneider A, Gonnet GH (2006). Fast estimation of the difference between two PAM/JTT evolutionary distances in triplets of homologous sequences. BMC Bioinformatics.

[B22] Schneider A, Dessimoz C, Gonnet GH (2007). OMA Browser – exploring orthologous relations across 352 complete genomes. Bioinformatics.

[B23] Benson DA, Karsch-Mizrachi I, Lipman DJ, Ostell J, Wheeler DL (2007). GenBank. Nucleic Acids Res.

[B24] Dayhoff M, Schwartz R, Orcutt B, Dayhoff M (1978). A model for evolutionary change in proteins. Atlas of Protein Sequence and Structure.

[B25] Farrar M (2007). Striped Smith-Waterman speeds database searches six times over other SIMD implementations. Bioinformatics.

[B26] Bjorklund AK, Ekman D, Light S, Frey-Skott J, Elofsson A Domain rearrangements in protein evolution. J Mol Biol.

[B27] Bateman A, Coin L, Durbin R, Finn RD, Hollich V, Griffiths-Jones S, Khanna A, Marshall M, Moxon S, Sonnhammer ELL, Studholme DJ, Yeats C, Eddy SR (2004). The Pfam Protein Families Database. Nucleic Acids Res.

[B28] Fulton D, Li Y, Laird M, Horsman B, Roche F, Brinkman F (2006). Improving the Specificity of High-throughput Ortholog Prediction. BMC Bioinformatics.

[B29] Dessimoz C, Boeckmann B, Roth ACJ, Gonnet GH (2006). Detecting non-orthology in the COGs database and other approaches grouping orthologs using genome-specific best hits. Nucleic Acids Res.

[B30] Balasubramanian R, Fellows M, Raman V (1998). An improved fixed-parameter algorithm for vertex cover. Information Processing Letters.

[B31] Gil M, Dessimoz C, Gonnet GH (2005). A dimensionless fit measure for phylogenetic distance trees. J Bioinform Comput Biol.

[B32] Notebaart RA, Huynen MA, Teusink B, Siezen RJ, Snel B (2005). Correlation between sequence conservation and the genomic context after gene duplication. Nucleic Acids Res.

[B33] Altenhoff AM, Dessimoz C (2008). Phylogenetic and functional assessment of Orthology Inference Projects and Methods.

[B34] OMA Browser. http://omabrowser.org.

